# Development and initial Experience of an online Exchange Platform on Sex and Gender Aspects in Medicine: “GenderMed-Wiki”

**DOI:** 10.3205/zma001178

**Published:** 2018-08-15

**Authors:** Julia Schreitmüller, Jan C. Becker, Daniel Zsebedits, Marvin Weskott, Mahboobeh Dehghan-Nayyeri, Christian Fegeler, Matthias Heue, Margarethe Hochleitner, Andrea Kindler-Röhrborn, Bettina Pfleiderer

**Affiliations:** 1 University of Muenster, Institute of Clinical Radiology, Muenster, Germany; 2 University of Muenster, Medical Faculty (IfAS), Department of Medical Education, Muenster, Germany; 3 Heilbronn University, GECKO Institute for Medicine, Informatics and Economics, Heilbronn, Germany; 4 University of Duisburg-Essen, Medical faculty, Essen, Germany; 5 Medical faculty Innsbruck, Gender Medicine Unit, Innsbruck, Austria; 6 University Hospital Duisburg-Essen, Institute of Pathology, Essen, Germany

**Keywords:** Gender medicine, sex/gender sensitive medicine, knowledge platform, gender, sex, medical teaching, medical study

## Abstract

**Goal: **Knowledge about sex/gender aspects in medicine is often lacking, even though this serves as base for individualized patient-centered care. Thus we developed an online exchange platform on sex and gender aspects in medicine: “GenderMed-Wiki” [www.gendermed-wiki.de]. This was funded by the German Federal Ministry of Education and Research (BMBF; FKZ: 01FP1506). Our goal is to facilitate the integration of sex and gender in all areas of medicine. Therefore we evaluated if "GenderMed-Wiki" is suitable to provide knowledge on sex and gender aspects in medicine adequately.

**Methods: **Qualitative evaluation of “GenderMed-Wiki” was done 6 months after project start by 4 focus groups with a total of 30 participants (students, lecturers, physicians, and the public). The discussions in each focus group were minuted, requirements pooled and new categories derived inductively. After further optimization of the platform a quantitative survey was done by an online questionnaire (SoSci Survey). 149 students of the medical faculties of Muenster and Duisburg-Essen (as well as students of dentistry from the medical faculty of Muenster) participated (return rate of 3.3%). Evaluation of the content of the articles was done by assessing three professional articles: Sex/gender and medicine (both study courses medicine and dentistry), depression (medicine only) and periodontitis (dentistry only). The results were reported in relative and absolute frequencies and associations were assessed by Chi-Quadrat-tests.

**Results: **Four categories which needed further optimization were deducted from the responses given by our focus groups prior to evaluation: *aspects related to content, technical requirements, usability of the platform and legal challenges*. Most of the students found “GenderMed-Wiki” to be informative, however they didn´t think it to be relevant for their current studies. In contrast, many thought that the platform may be useful when working as physicians.

Students who reported that topics related to sex and gender were not of importance to them, evaluated the platform more neutrally and answered questions related to sex/gender in depression more often incorrectly.

**Conclusions: **Focus groups are a useful approach to identify necessary changes in projects in a systematic way. After further optimizations, “GenderMed-Wiki” seems to be suitable to facilitate the integration of sex/gender into medical teachings. It is of importance, however, to change the attitude of students towards sex/gender sensitive medicine (e.g. by integration into the medical curriculum), since this influences strongly how this platform is perceived and how someone deals with its contents.

## Zusammenfassung

**Zielsetzung:** Wissen um Geschlechteraspekte in der Medizin ist meist ungenügend vorhanden, obwohl sie die Grundlage für eine individualisierte Versorgung darstellen. Daher wurde in einem BMBF geförderten Projekt (FKZ: 01FP1506) eine online Austauschplattform GenderMed-Wiki [www.gendermed-wiki.de] zu Themen um Geschlecht und Medizin aufgebaut. Ziel ist die Vermittlung von Fachwissen zu Geschlechteraspekten in allen Bereichen der Medizin. Evaluiert wurde deshalb u. a., ob GenderMed-Wiki geeignet ist, Wissen zu Geschlecht und Medizin adäquat zu vermitteln.

**Methodik: **In Form von vier Fokusgruppen mit insgesamt 30 Teilnehmenden (Studierende, Lehrende, ÄrztInnen, Öffentlichkeit) fand eine qualitative Evaluation von GenderMed-Wiki sechs Monate nach Projektbeginn statt. Die Diskussionen wurden protokolliert, ein Anforderungspool gebildet und induktiv Kategorien abgeleitet. Nach Optimierung der Plattform wurde eine quantitative Evaluation per Onlinefragebogen (Sosci Survey) durchgeführt. Es nahmen 149 Studierende der medizinischen Fakultäten Münster und Duisburg-Essen (sowie der Zahnmedizin Münster) teil (Rücklaufquote: 3.3%). Die inhaltliche Konzeption der Plattform wurde anhand von drei Fachartikeln evaluiert: Geschlecht und Medizin (beide Studiengänge), Depression (Humanmedizin) und Parodontitis (Zahnmedizin). Die Ergebnisse wurden als relative und absolute Häufigkeiten dargestellt, Zusammenhänge wurden mittels Chi-Quadrat-Test ermittelt.

**Ergebnisse: **Mit Hilfe der Fokusgruppen wurden vier Kategorien mit Änderungsbedarf abgeleitet und teilweise für die Studierendenbefragung optimiert:* Inhaltliche Aspekte, Technische Anforderungen, NutzerInnenpraxis* und* Rechtliche Herausforderungen*. Viele Studierende bewerten GenderMed-Wiki als lehrreich, hielten die Plattform jedoch v. a. für ihren späteren beruflichen Alltag und weniger für ihr Studium für relevant. Studierende, die geschlechtersensible Themen weniger bedeutend finden, bewerteten die Plattform eher als neutral und beantworteten Fragen zu Geschlecht und Depression häufiger falsch.

**Schlussfolgerung: **Fokusgruppen sind hilfreich, um projektbezogenen Änderungsbedarf systematisch herauszuarbeiten. Nach entsprechender Anpassung scheint GenderMed-Wiki gut geeignet, um die Integration von Geschlecht in die medizinische Lehre zu fördern. Wichtig ist es, die Einstellung der Studierenden zur geschlechtersensiblen Medizin zu verändern (z. B. durch Integration in das Studium), denn diese bedingt auch wie man die Plattform wahrnimmt und wie gut man sich mit ihr auseinandersetzt.

## 1. Introduction

Sex/gender aspects in medicine were considered to be of minimal importance for a long time and medical knowledge was mostly based on studies in males. Recent studies have demonstrated, however, that incidence, symptomatology and course of many diseases are influenced by sex and gender [1], [2], [3], [4]. Thus it is of importance to take biological (sex) and socio-cultural (gender) aspects as next steps towards an individualized medicine into account.

Currently, knowledge about sex and gender sensitive medicine is neither sufficiently present nor ordered in a systematic way [4]. Consequently, physicians do not integrate sex/genders aspects in their daily clinical practice, leading to potentially incorrect or delayed diagnoses and suboptimal therapies for men and women. Heart attacks in women have been often diagnosed too late, since they depict “unspecific” symptoms like pain jaw or back pain and/or vasovagal symptoms. In addition, myocardial infarction is still too often considered to be a “male disease” which occurs less frequent in women [5], [6]. Osteoporosis, in contrast, is being looked at as a typical “female disease” in a stereotypical way and is therefore underdiagnosed and under-researched in men. In that vein, young women were used as reference group when assessing bone density in elderly men [7]. 

**Figure 1:**
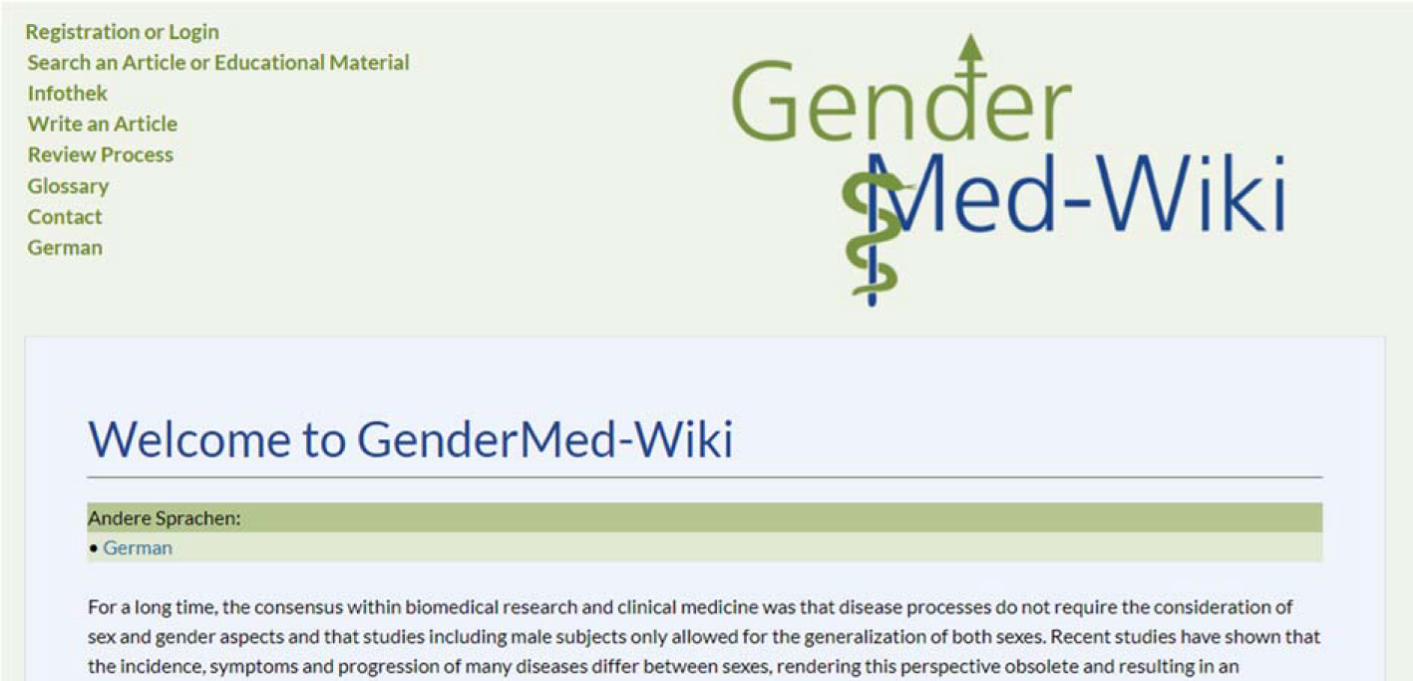
Homepage of “GenderMed-Wiki” [https://www.gendermedwiki.uni-muenster.de/mediawiki_en]

A paradigm shift in medicine is needed to integrate sex and gender and its interaction in treatment of patients and research concepts [8], [9]. Sex/gender contents need to be taught in medical studies for a successful integration [10]. We could demonstrate in a previous research project of the medical faculties Muenster and Duisburg-Essen (funded by the German Federal Ministry of Education and Research and the European social fund of the European Union (FKZ: 01FP1101)) that knowledge about sex/gender differences and related impact in prevention and treatment of patients was not sufficiently present [11]. Therefore we decided to develop in a follow-up project a national online exchange platform on sex and gender aspects in medicine “GenderMed-Wiki”, which is based on the principles of a Wiki. “GenderMed-Wiki” is a joint initiative of the medical faculties in Muenster, Duisburg-Essen and Innsbruck, as well as the GECKO Institute for Medicine, Informatics and Economics of Heilbronn University. The platform can be reached via the link www.gendermed-wiki.de (see Figure 1 (Fig. 1)).

## 2. Project description

### 2.1. “GenderMed-Wiki” as interactive, digital process

As consequence of the increasing digitalization it is necessary to find new ways of knowledge generation in medicine. Collective sharing of data as main principle of a Wiki encompasses innovative processes like co-creation and data sharing [12], [13], [14]. At the one hand, “GenderMed-Wiki” presents the unique opportunity to obtain actual information on sex/gender in medicine; on the other hand experts (scientists, physicians in clinical practice, students) can provide new content or share expert knowledge with others in the community (data sharing). It is also possible to work with other authors together on the same content (co-creation). By this, articles will be continuously updated and sex/gender specific knowledge collated by various experts. By using the wisdom of crowds, exponential increase of knowledge of sex/gender aspects in medicine will be facilitated. All articles will be peer-reviewed prior to being published by an interdisciplinary review board of experts in sex/gender sensible medicine as well as by 2 editors (members of the “GenderMed-Wiki” team). “GenderMed-Wiki” will serve as digital scientific bridge towards sex/gender sensitive medicine. 

**Figure 2:**
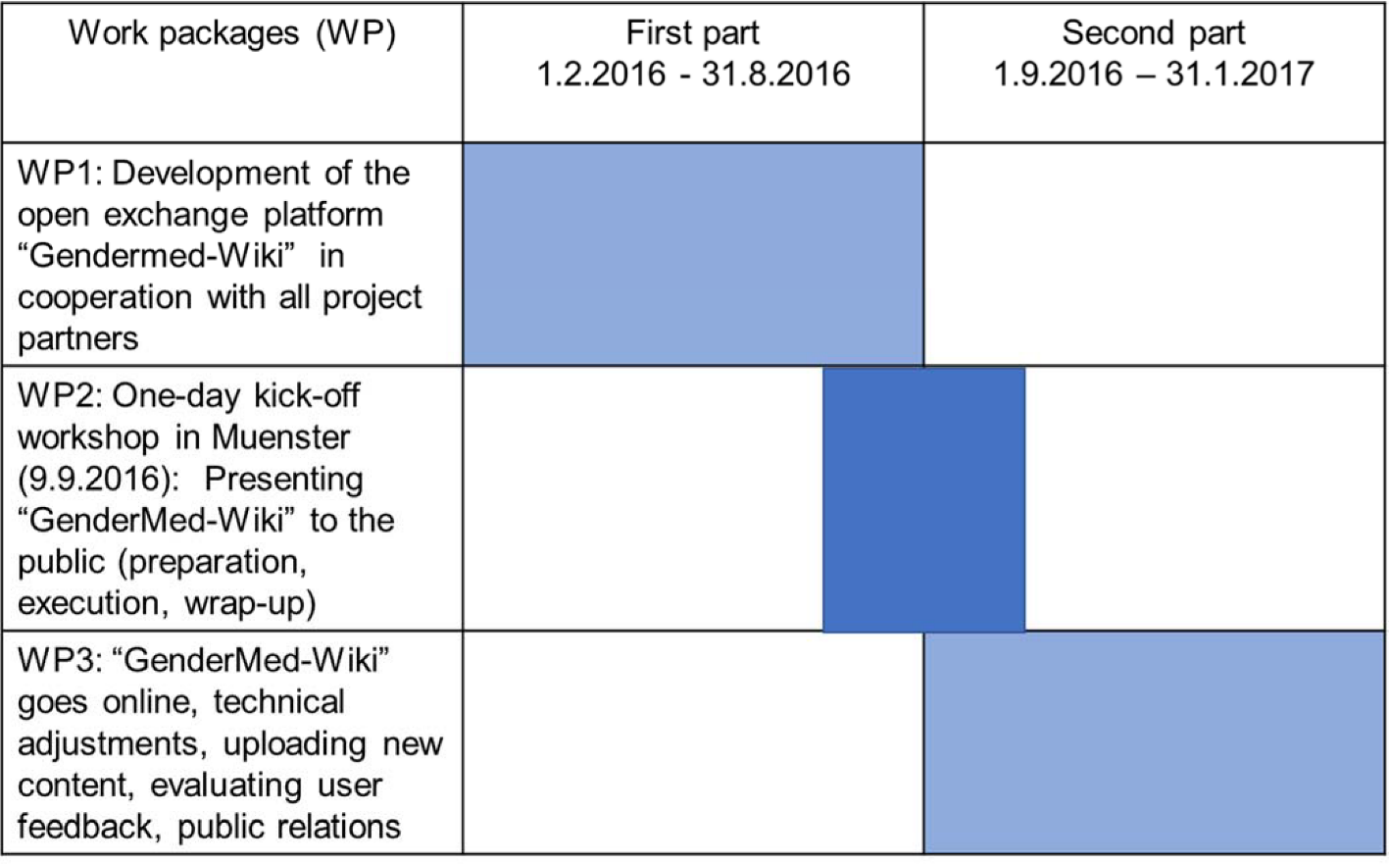
Project development (timeline) with three work packages.

**Figure 3:**
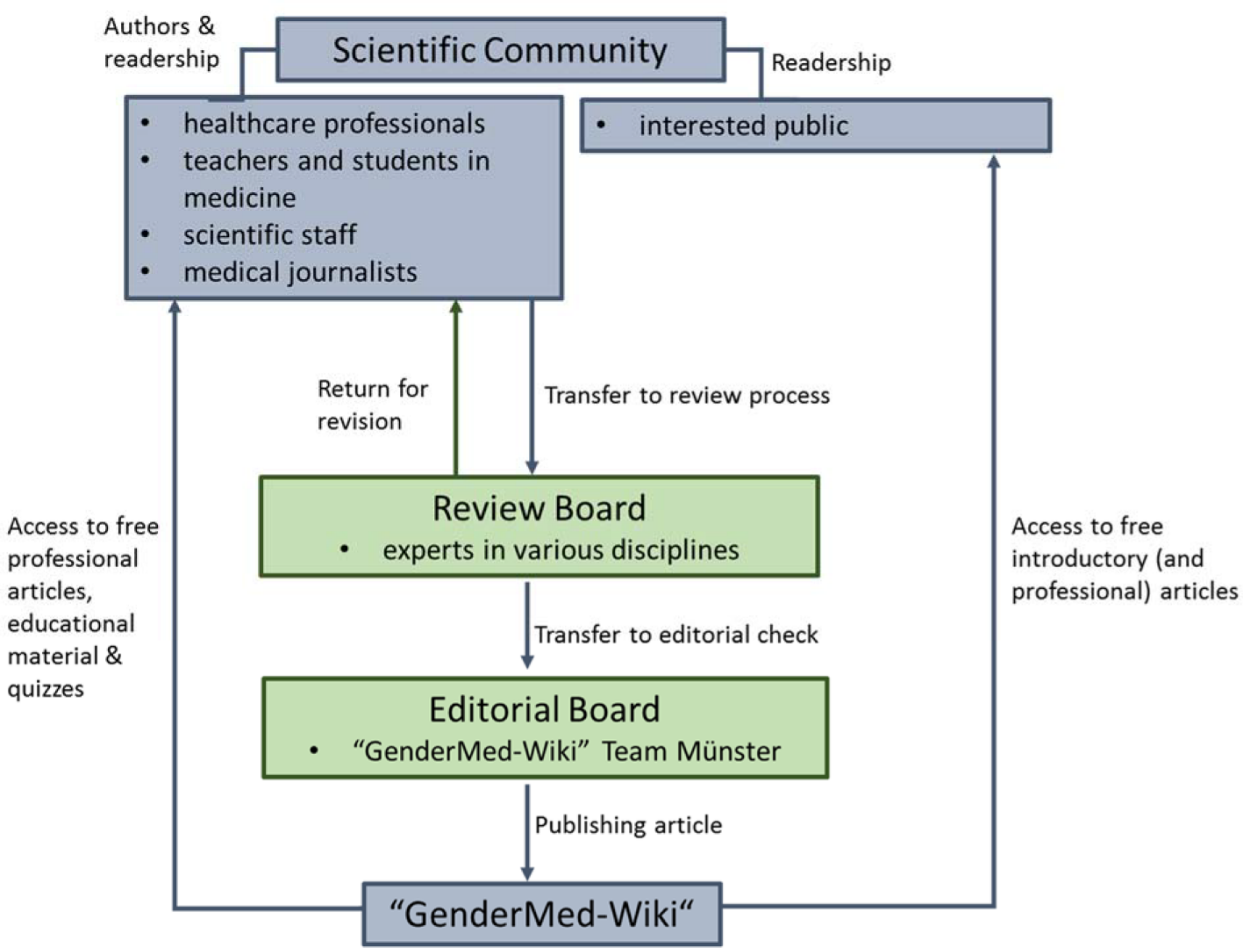
Functional processes of “GenderMed-Wiki”.

To reach this goal an interdisciplinary team of computer scientists, medical doctors as well as other experts from fields such e.g. psychology or biology worked closely together for one year. In addition, a board of specialists on sex/gender from medical and non-medical areas was formed at project start, who agreed to continue supporting “GenderMed-Wiki” after the funding of the project ended. The development of the project is shown in Figure 2 (Fig. 2) and detailed functional processes of the Wiki are depicted in Figure 3 (Fig. 3). The project was funded from 1^th^ of February 2016 – 31^th^ of January 2017.

#### 2.2. Goals of “GenderMed-Wiki”

“GenderMed-Wiki” serves as a national exchange- and knowledge platform on sex/gender aspects in medicine. Combined expert knowledge on sex/gender differences in disease and related therapy will be made available online and will be amended by interaction with the “scientific community”. Sex/gender sensitive articles (professional articles and article for the public) and teaching materials (e.g. presentations and case studies) from all areas of medicine will be shared free of charge. Goal is to facilitate the integration of sex/gender aspects into medicine and medical teachings. “GenderMed-Wiki” will provide professional up-to date information for various actors in the health system (e.g. physicians, representatives of health insurances) and researchers. Teaching materials can be integrated directly as add-on into the medical curriculum, as well. 

#### 2.3. Evaluation of the project 

##### 2.3.1. Qualitative evaluation by focus groups

“GenderMed-Wiki” was officially launched in Muenster at the 9th of September 2016. Contents and usability of the platform was presented in form of a one day kick-off workshop to the public. The status-quo of the project and future perspectives was demonstrated by input-talks in the morning. With regard to contents and structures, 4 interdisciplinary focus groups with a total of 30 participants (f=19, m=11) met in the afternoon and discussed further needs of optimization. The focus groups were formed based on the different competences of the participants: medical students and subjects with a profession close to medicine (n=7), lecturers in medicine as well as lectures in subjects close to medicine (n=8), physicians (n=7) and the public (e.g. gender mainstreaming, press; n=8). 

All participants were asked to assess two articles prior to the workshop: one article either on sex/gender and depression or itch and the other on sex/gender and medicine to facilitate discussion. The focus groups were chaired by 2 members of the interdisciplinary review board. Chairs were briefed in advance by additional information sent by e-mail. The discussion was moderated in a structured way by use of a field manual and topics such as sustainability, incentives for readers and authors to use the platform and missing items in the platform were addressed. The goal of these focus groups was to identify further optimization steps of the platform prior to their quantitative assessment. Minutes of the discussions within each focus group were kept, requirements pooled and new categories derived inductively.

##### 2.3.2. Quantitative evaluation of the project by students

After further optimization of the platform, its usability was evaluated in a pilot study by student samples from the medical faculties of Muenster and Duisburg-Essen (as well as dentistry students of the medical faculty Muenster). Students from both faculties were informed about “GenderMed-Wiki” by using faculty e-mail lists and were invited to participate in the evaluation of the new platform. The platform was not part of the medical curriculum at that time. To increase motivation to participate in the survey, beside the opportunity to be one of the first to use innovative teaching materials, book tokens were drawn. 

One of the main research questions was to assess whether “GenderMed-Wiki” was judged by students (as important user group) to be a suitable tool to obtain sex/gender information in medicine. We were also interested to learn more about the personal opinion of the participants on sex/gender topics in medicine in general and about their perceived individual knowledge level on sex/gender aspects. This was done by evaluation of three professional articles, which were already completed at that time and were peer-reviewed as well: sex/gender and depression (medical students of both faculties), module on sex/gender and medicine (medical students of both faculties) and sex/gender in periodontitis (dentistry students, medical faculty of Muenster). The professional articles were completed by additional teaching materials such as a presentation and a case study. Evaluation was done by an online questionnaire. The questionnaire was drafted with the online tool “SoSci oFb” [https://www.soscisurvey.de/]. The design of the questionnaire was based on the paper of Burghaus et al. (2016) (Gender-specific Aspects of Knowledge and Gender Sensitivity in Medical Education – An Inventory). The questionnaire was further optimized by an interdisciplinary team of psychologists, physicians, dentists and natural scientists. The questionnaire consisted of 55 items with mainly using 5-point-Likert-scales (from -2 to +2) as response categories. Contents were clustered according to 6 themes:

1. Characteristics of participants (7 items, e.g. sex, age, number of years studying at medical school),
2. Knowledge about sex/gender aspects in medicine (15 items, e.g. *When treating male or female patients does it make a difference whether male or female patients are treated by male or female physicians?*),
3. Perceived one´s own competence on gender medicine and relevance of these aspects in medicine (5 items, e.g. *How relevant is it for you that sex/gender aspects will be integrated in medical teachings?*),
4. Knowledge quiz (5 items related to the module on sex/gender and medicine and sex/gender in depression, e.g. *Which of the following definitions of gender is correct?*),
5. Evaluation of the professional articles (3 items related to the module sex/gender and medicine, 5 items related to depression, e.g.* Is the outline of the article clear?*),
6. Evaluation of the platform (10 items, e. g. *Is the platform easy to use?*).

The questionnaire was sent to 3934 medical students und 604 dentistry students by e-mail. The evaluation period was from 24.10.2016 till 30.11.2016. Results were reported in absolute and relative frequencies, and associations were assessed by Chi-square-tests.

## 3. Results

### 3.1. Focus groups

**Figure 4:**
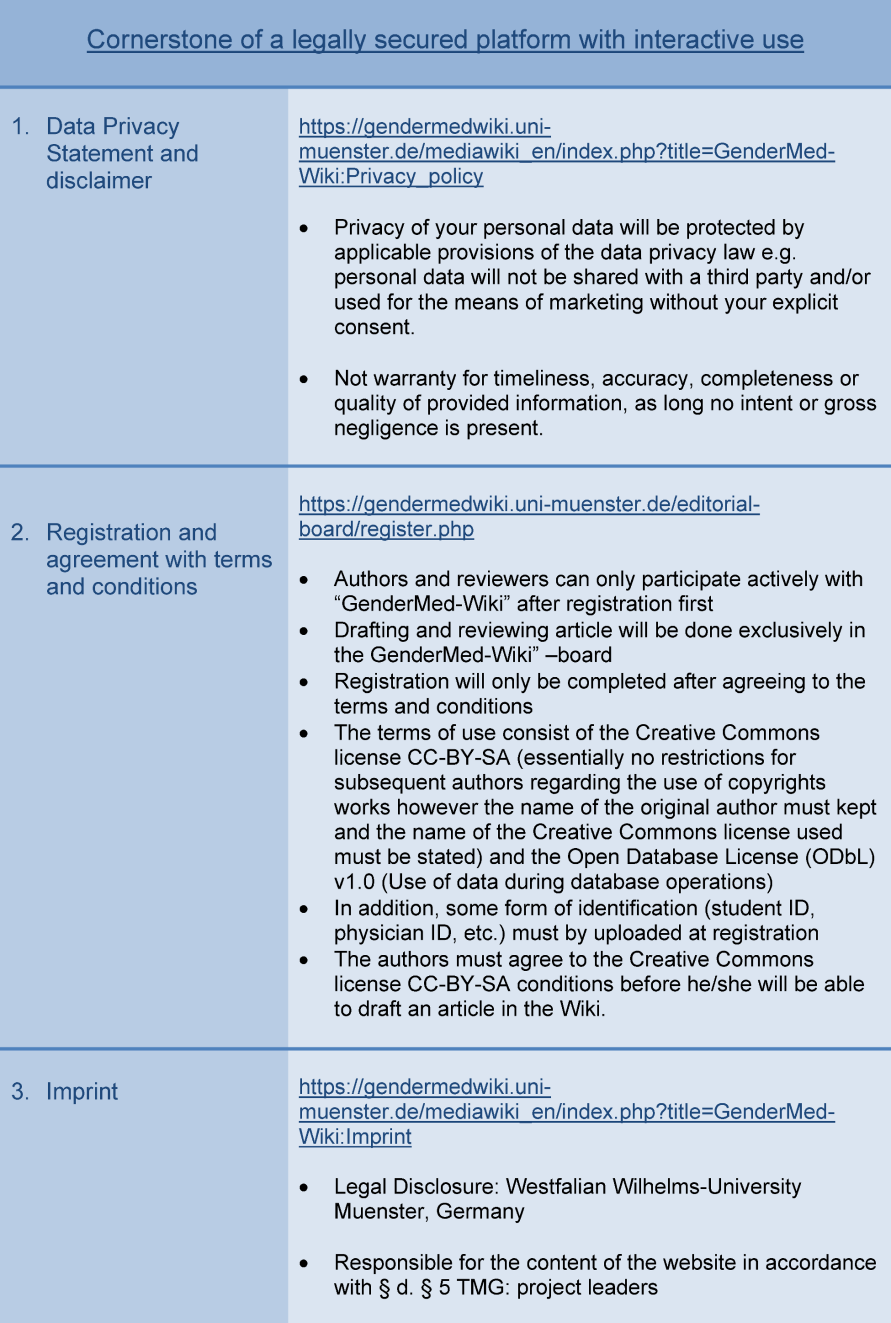
Legal requirements of an interactive platform with different user groups (version: February 2018).

**Figure 5:**
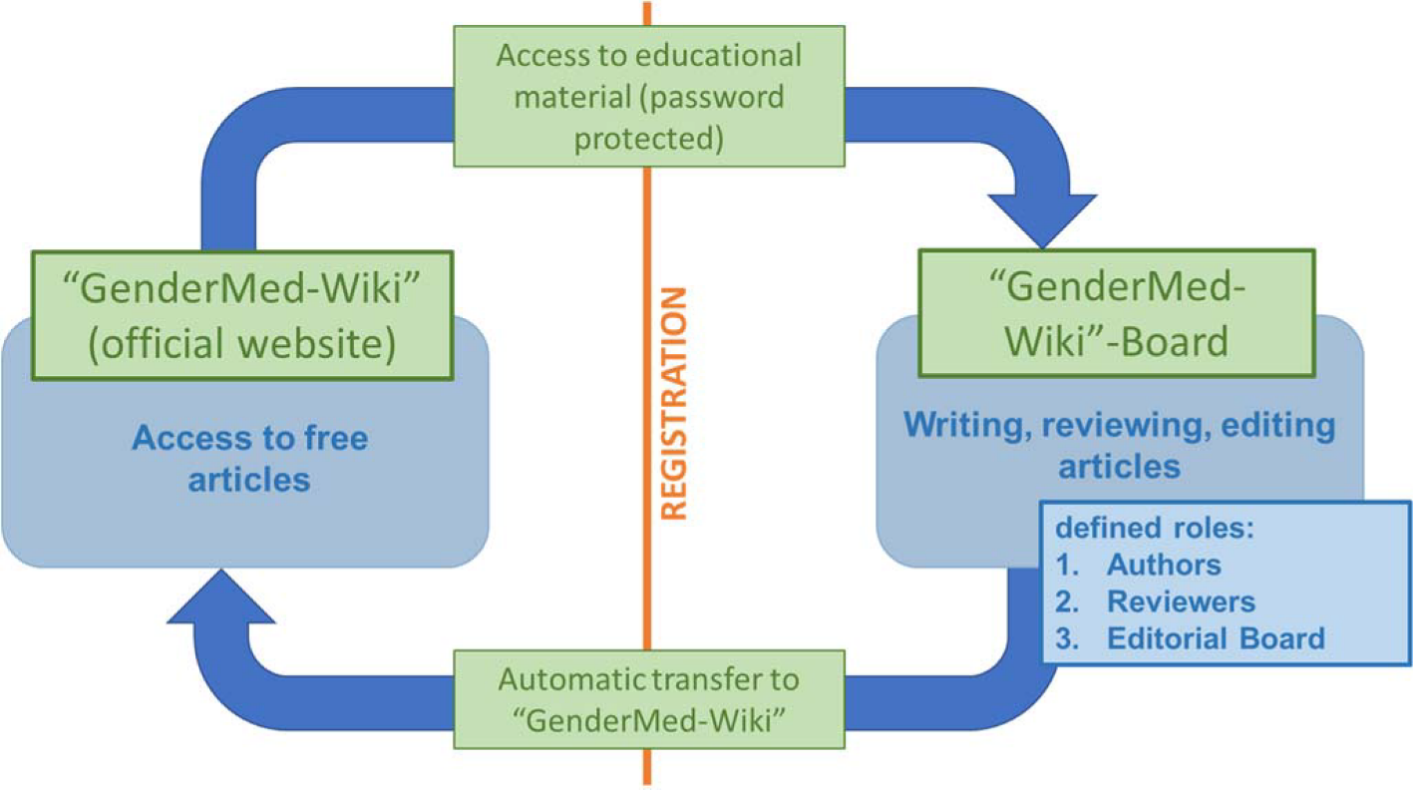
Functional IT architecture of “GenderMed-Wiki” with two features. Adaption of the technical processes to comply with legal requirements.

Four categories were identified which needed further optimization and those were integrated accordingly (see Attachment 1):

- *Aspects related to content*: e.g. how easy was it to download abstracts, quality of introductory articles (fact-sheets) and professional articles, integration of a glossary and development of quizzes for all articles.
- *Technical requirements*: e.g. PDF- function for all articles.
- *Usability of the platform*: e.g. it was suggested to offer an elective study course on gender medicine. Part of the academic assessment could be drafting an article for “GenderMed-Wiki”. In case this will be proven to be a suitable format, this could be used at other universities as well to increase the acceptance of “GenderMed-Wiki” in the student population.
- *Legal challenges*: e.g. data protection and copyright issues, disclaimers being different for different user groups (readers, authors and reviewers; see Figure 4 (Fig. 4)). To take these entire legal requirements into account, the IT architecture of this platform was adapted accordingly (see Figure 5 (Fig. 5)).

#### 3.2. Study sample

149 students participated in the survey, of which 119 were medical students. The return rate was 3.3% in total and 3% for medical students. The following results are based on the sample of the medical students only. The characteristics of the study sample are shown in table 1 (Tab. 1). 67% (80/119) studied at the medical faculty of the University of Muenster and 29% (35/119) at the medical faculty of the University of Duisburg-Essen.

##### 3.2.1. Relevance of sex/gender sensitive medicine

The analyses of our questionnaire revealed that the following 5 subjects were thought to have the highest sex/gender relevance: *general medicine* (61%),* psychosomatic medicine and psychotherapy* (46%), *anatomy* (45%), *internal medicine* (36%) and *urology *(29%). In contrast,* dentistry, clinical environmental medicine, clinical-pathological conferences, infectiology and immunology* were thought to have the lowest sex/gender relevance (each received one vote only). Participants could choose 5 subjects with the highest sex/gender relevance from a drop-down list of preclinical, clinical and interdisciplinary subjects. Students were mostly in agreement that sex of a patient has an important impact on patient care: sex/gender differences were seen most relevant related to the frequency of preventive medical check-ups and related to strategies of coping with one´s disease. Sex/gender differences in the presentation of symptoms, expectations related to medical care and in particular sex of the physician treating patients were considered to be less important (see table 2 (Tab. 2)). No sex differences were seen in the frequency of the responses given.

##### 3.2.2. Sex/gender sensitive competence of students and relevance of the platform 

Most students assumed having a low competence in sex/gender aspects in medicine (item: *How would you rate your own competence in sex/gender aspects in medicine?*): only 14% (17/119) reported having a good or very good competence. To obtain a more objective measure of student´s knowledge increase after reading the exemplary articles on depression and the module on sex/gender in medicine, a quiz related to the topics of the articles had to be completed. None of the participants were able to answer all 5 questions about depression and sex/gender correctly. 3% (2/72) of students being in the clinical part of medical training gave one incorrect answer, while students being in the preclinical part made at least 2 mistakes. 32% (8/25) preclinical students and 44% (32/72) of students being in the clinical part made no mistakes when answering questions related to the module sex/gender in medicine. 12% (3/25) of preclinical students and 8% (6/72) of clinical students were not able to answer any of the questions correctly (see table 3 (Tab. 3)).

63% (64/102) of those responding judged “GenderMed-Wiki” as being relevant or very relevant when working later as physician (item: *Do you think that “GenderMed-Wiki” will be relevant for your future clinical practice?*). 28% (28/102) thought that “GenderMed-Wiki” will be relevant or very relevant for their medical studies (item: *How relevant is “GenderMed-Wiki” for your medical studies?* see table 4 (Tab. 4)). Students, who considered “GenderMed-Wiki” as being relevant or very relevant when working later as physician, also thought that the platform would be relevant for their medical studies (Χ^2^ (16, *N*=102)=59.64, *p*<0.0001). Students, who did not believe that the platform would be useful when working later as physician, made more mistakes when answering questions related to sex/gender and depression (Χ^2^ (16, *N*=86)=31.32, *p*=0.012).

##### 3.2.3. Evaluation of the platform 

Results of chi-square-tests indicated that students who didn´t think that “GenderMed-Wiki” would be relevant when working later as physician, evaluated the platform more neutrally (or negatively). An association between the relevance of “GenderMed-Wiki´s” future usability and usability of the platform (Χ^2^ (16, *N*=101)=47.94, *p*<0.0001), comprehensibility of the case studies (Χ^2^ (8, *N*=99)=21.78, *p*=0.005) and presentations (Χ^2^ (12, *N*=101)=26.15, *p*=0.01), as well as ease of use (Χ^2^ (12, *N*=100)=29.46, p=0.003) was observed. Those who considered the platform to be relevant when working as a physician evaluated the professional articles as being more useful (Χ^2^ (16, *N*=102)=89.15, *p*<0.0001). No sex differences were seen in the frequency of the responses given.

## 4. Discussion

Discussions within the focus groups identified 4 categories which needed further optimization: *aspects related to content, technical requirements, usability of the platform and legal challenges.* Aspects related to content e.g. such as restructuring of the page layout and aspects related to technical requirements such as e.g. the integration of a pdf-creation tool for all articles were implemented prior to the evaluation of the platform by student users. Optimizations regarding the usability of the platform (e.g. implementation of “GenderMed-Wiki” in the medical curriculum) and legal challenges (e.g. data protection issues) were addressed in the second part of the project (work package 3) paralleling the evaluation phase. 

Discussions in focus groups are a feasible and useful approach to facilitate an intensive thematically-driven exchange within groups and to discuss and analyze items by taking into account different perspectives. This enabled us to identify different categories of great importance to reach our project goals: e.g. it was necessary to adapt the technical processes to comply with legal requirements. As a consequence, two parallel platforms were installed and information is automatically transferred from one platform to the other. “GenderMed-Wiki” and all articles are online freely available to everyone, in contrast, reviewers and authors can only access and write articles in the platform “GenderMed-Wiki” board after registration and proof of identity (e.g. student ID or employer ID). In the same vein, teaching materials are also stored in the “GenderMed-Wiki” board and can only be seen and downloaded after registration and log-in (see Figure 5 (Fig. 5)).

Students evaluated the platform after optimization as being relevant for their future medical practice. So far, these aspects are not part of the regular medical curriculum. Remarkably, students considered sex/gender aspects in medical studies as being relevant, but felt at the same time that their knowledge and competence is very low. This corroborates findings of a previous study of Burghaus et al. (2016). The authors could show that knowledge about sex/gender aspects in medicine is not sufficiently present in students, lecturers and professors to adequately address the impact of sex and gender on prevalence of disease, prevention, diagnosis and treatment of patients [11]. Therefore, it is not surprising that the students of this study sample did not believe that sex and gender is of importance in subjects like *infectiology* and *immunology.* This is in contrast to findings of many research studies who reported that females have a more effective immune response towards viruses and other pathogens, since estrogens are able to stimulate the production of IgG and IgM antibodies. The more effective immune response of women as compared to men leads at the same time to a much higher frequency of autoimmune diseases in women [8]. 

The case studies and presentations of “GenderMed-Wiki” were rated by most students as being informative and useful. Structure and contents of the platform seems to be well suited to teach sex/gender sensitive knowledge adequately. Those students who did not believe that sex/gender aspects are relevant in medicine evaluated the platform (articles and teaching materials) neutrally more often and made more mistakes when answering questions related to sex/gender and depression in the knowledge quiz. Subjects often favor neutral responses if they have a choice. This phenomenon is known as “tendency towards the middle” in psychology. Reasons for this behavior, in addition to having a true neutral opinion, are insecurity, not having enough knowledge, low openness and lack of motivation [15]. Participation at the evaluation required reading of two long articles necessitating a high level of motivation of the students. The results seem to indicate that those with a lower motivation and less interest in sex/gender questions in medicine seemed not to read the articles in full detail and made significantly more mistakes in the quiz on sex/gender and depression. Taken this all together, those students who don´t believe that gender medicine is important did not keep so busy with the contents of the platform and therefore did not gain as much knowledge after reading the articles and made more mistakes in the knowledge quizzes. It may be feasible to speculate that students who rated the platform more neutrally were less open towards sex/gender sensitive themes and less motivated to acquire new competences in this topic. However, one needs to keep in mind that at the time of the evaluation “GenderMed-Wiki” was not part of a medical curriculum und participation of the evaluation was voluntarily. The return rate of 3.3% was very low as well and the responses are not by any means representative. They are hints at best and this is certainly a limitation of the current study. 

Nevertheless, the results confirm that it is necessary to motivate and sensitize students to deal with sex/gender contents in medicine, foster interest and increase knowledge about this topic. To increase motivation (at first extrinsically), sex/gender aspects must be relevant in written and oral exams. Often items are only considered to be relevant, if they are being part of exams and grades (and therefore being in part rewarded) [16], [17]. Without any change in motivation and sensitization for sex/gender aspects in medicine (e.g. symptomatology), important input may be missed and in the worst case diseases are not diagnosed and treated adequately [9].

“GenderMed-Wiki” is an important step towards a sex/gender sensitive medicine and full integration into medical studies. To reach this goal it is important to include lecturers in preclinical and clinical subjects in the debates. Without acceptance of the faculty it will be too difficult to integrate teaching materials such as presentation and case studies of the platform in medical lectures. In a next step, “GenderMed-Wiki” could be evaluated by the lecturers and professors using it. This would not only help us in further optimization of the platform, but it may also stimulate its acceptance and sensitize for integrating sex/gender aspects into medical education. 

Taken into account international standards [18], only one medical faculty in Germany integrated sex/gender sensitive themes successfully in their medical curriculum and only a few medical faculties offer this as an elective course [10], [19]. Sex/gender aspects should be part of all teaching materials from preclinical to clinical modules. Sex and gender as well its interactions should be taught. A successful and necessary strategy to fully integrate this in the medical curriculum systematically and interdisciplinary is to appoint a change agent (consultant in organizational transformative processes) [19]. According to Ludwig et al (2016), integration of these aspects in German medical curricula may be achieved by the NKLM (National competence oriented teaching catalogue in medicine) [10].

One of the main challenges of “GenderMed-Wiki” is not only to increase the number of users but also to add new articles to the platform. “GenderMed-Wiki” should therefore cooperate closely with other medial faculties. Students could be sensitized by elective courses on gender medicine (in Muenster successfully implemented since summer 2017), by integrating sex/gender aspects into their doctoral dissertations or by drafting new articles for “GenderMed-Wiki”. This could result in an increase in competence and knowledge and could foster the acceptance and integration of the online exchange and knowledge platform “GenderMed-Wiki” into medical studies. 

## 5. Conclusions

Using focus groups was a good approach to identify necessary project related changes in “GenderMed-Wiki” in a systematic way. 

We were able to optimize the development processes of the platform by interdisciplinary debates and qualitative analyses of feedback. This was evaluated by an online questionnaire in a student sample. Our results confirmed that existing knowledge in sex/gender medicine is not known sufficiently und teaching materials are lacking. Sex/gender aspects are often not integrated at most German medical faculties. Students believe that sex/gender medicine is important (in particular when they will work as physician in clinical practice), but at the same time feel that they lack competence in this topic. The goal of “GenderMed-Wiki” is to stimulate the integration of sex/gender aspects in medicine. To achieve this goal is of importance to change the attitudes of many students; the more positive this topic is seen, the more willingly platforms such as “GenderMed-Wiki” will be used. A long-term goal is the full integration of sex/gender aspects into the medical curriculum. To foster sensitization and to increase the level of knowledge, “GenderMed-Wiki” can serve as an important intermediate step. One major challenge of “GenderMed-Wiki” is to keep the platform alive and to win experts from various disciplines as authors to continue to provide an up-to-date collection of articles on many relevant topics. 

## Funding

The project Development of an Exchange Platform “GenderMed-Wiki” is funded by the Federal Ministry of Education and Research (BMBF) under the project number 01FP1506. 

## Competing interests

The authors declare that they have no competing interests. 

## Supplementary Material

Minutes of the focus groups: four categories were identified which needed further optimization. Suggestions that were not implemented prior to the evaluation of the platform are marked in italic and bold.

## Figures and Tables

**Table 1:**
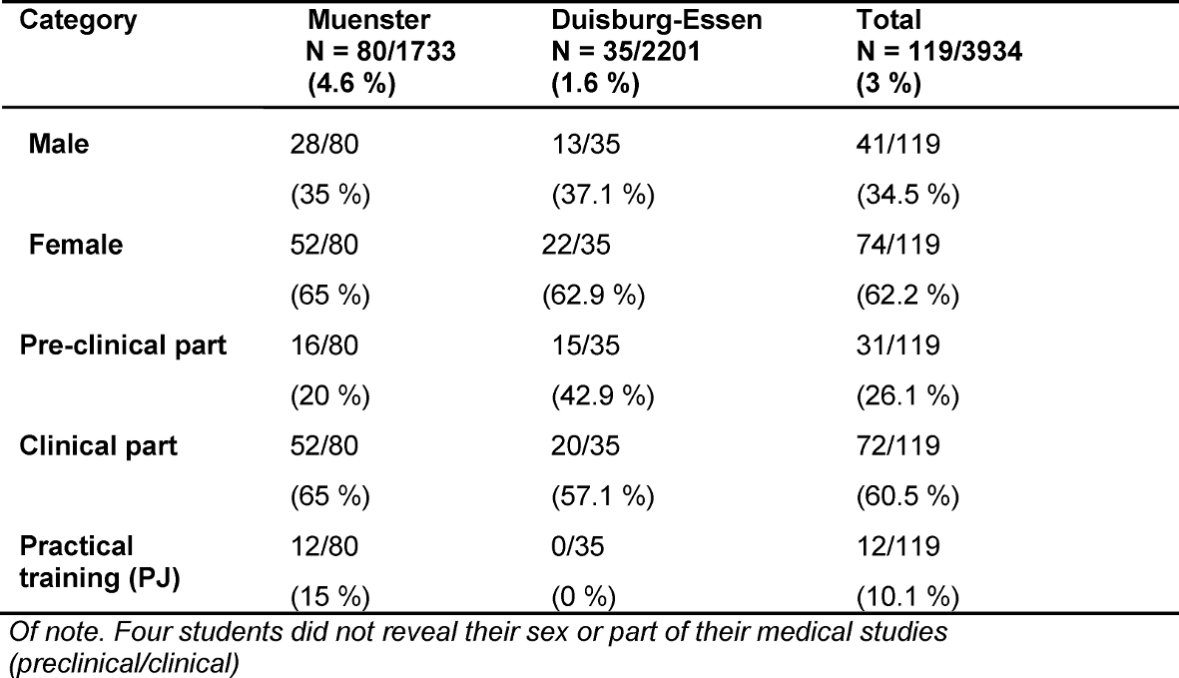
Characteristics of the study sample (medical students). Absolute and relative frequencies are shown.

**Table 2:**
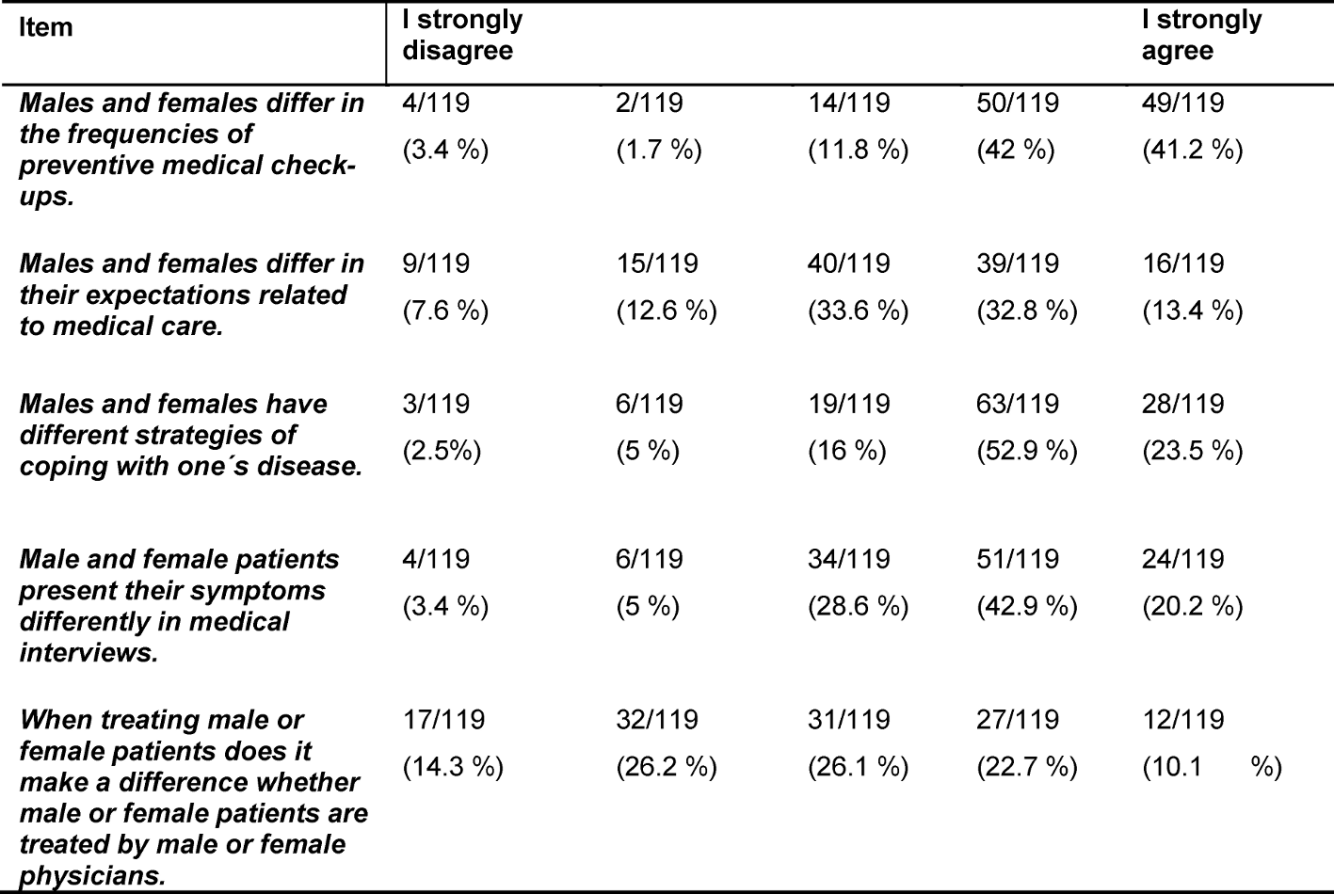
Responses are stratified according to sex. Absolute and relative frequencies are shown, N=119.

**Table 3:**
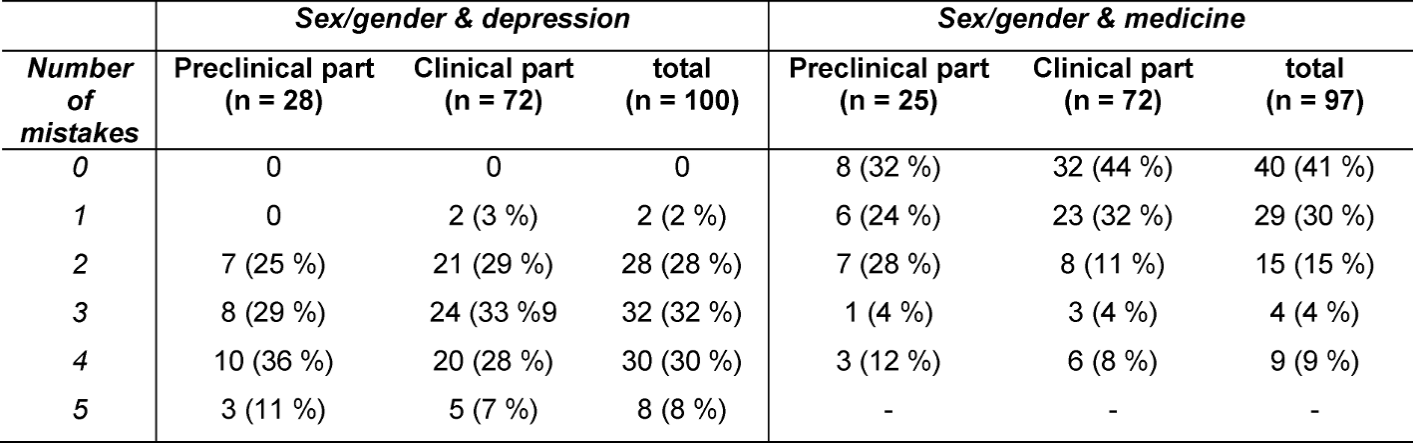
Number of mistakes made in the knowledge quiz on “sex/gender & depression” (N=100) and in the knowledge quiz on “sex/gender & medicine” (N=97). Absolute and relative frequencies are shown.

**Table 4:**
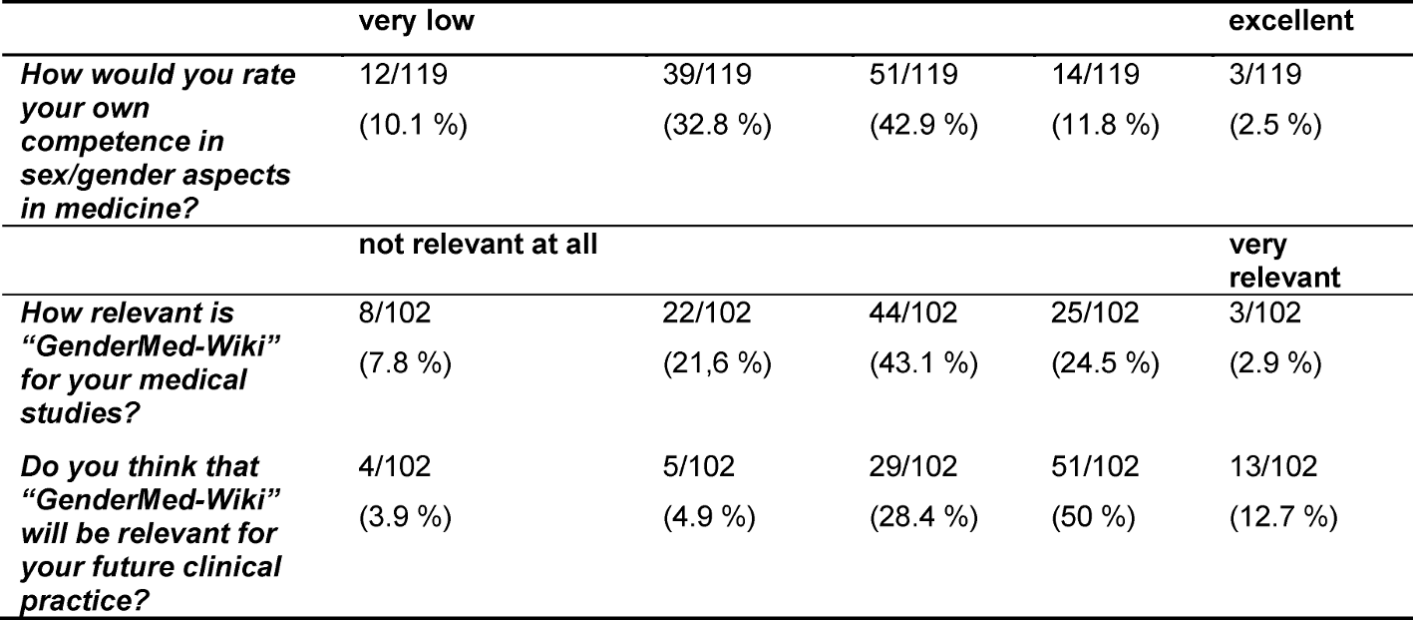
Rating of one’s own competence regarding sex/gender aspects in medicine and relevance of “Gendermed-Wiki” when working as a physician in the future. Absolute and relative frequencies are shown, N=119.

## References

[1] Fillingim RB, King CD, Ribeiro-Dasilva MC, Rahim-Williams B, Riley JL (2009). Sex, gender, and pain: a review of recent clinical and experimental findings. J Pain.

[2] Mittelstrass K, Ried JS, Yu Z, Krumsiek J, Gieger C, Prehn C, Roemisch-Margl W, Polonikov A, Peters A, Theis FJ, MEitinger T, Kronenberg F, Weidinger S, Wichmann HE, Suhre K, Wang-Sattler R, Adamski J, Illig T (2011). Discovery of sexual dimorphisms in metabolic and genetic biomarkers. PLoS Genet.

[3] Ober C, Loisel DA, Gilad Y (2008). Sex-specific genetic architecture of human disease. Nat Rev Genet.

[4] Pinn VW (2003). Sex and Gender Factors in Medical Studies. JAMA.

[5] Saner H (2007). Manifestation und Verlaufe der koronaren Herzkrankheit bei Mannern und Frauen--Konsequenzen fur Diagnose und Therapie. Therapeutische Umschau. Revue therapeutique.

[6] Mosca L, Banka CL, Benjamin EJ, Berra K, Bushnell C, Dolor RJ, Ganiats TG, Gomes AS, Gornik HL, Gracia C, Gulati M, Haan CK, Judelson DR, Keenan N, Kelepouris E, Michos ED, Newby LK, Oparil S, Ouyang P, Oz MC, Petitti D, Pinn VW, Redberg RF, Scott R, Sherif K, Smith SC SC, Sopko G, Steinhorn RH, Stone NJ, Taubert KA, Todd BA, Urbina E, Wenger NK, Expert Panel/Writing Group, American Academy of Physician Assistants, American Association for Clinical Chemistry, American Association of Cardiovascular and Pulmonary Rehabilitation, American College of Chest Physicians, American College of Emergency Physicians, American Diabetes Association, American Geriatrics Society, American Society for Preventive Cardiology, American Society of Echocardiography, American Society of Nuclear Cardiology, Association of Women's Health, Obstetric and Neonatal Nurses, Global Alliance for Women's Health, Mended Hearts, Inc, National Black Nurses Association, National Black Women's Health Imperative, National Women's Health Resource Center, North American Menopause Society, Partnership for Gender-Specific Medicine at Columbia University, Preventive Cardiovascular Nurses Association, Society for Vascular Medicine and Biology, Society for Women's Health Research, Society of Geriatric Cardiology, Women in Thoracic Surgery, WomenHeart: the National Coalition for Women with Heart Disease (2007). Evidence-based guidelines for cardiovascular disease prevention in women: 2007 update. J Am Coll Cardiol.

[7] Orwig DL, Chiles N, Jones M, Hochberg MC (2011). Osteoporosis in men: update 2011. Rheum Dis Clin North Am.

[8] Kindler-Röhrborn A, Pfleiderer B (2012). Gendermedizin - Modewort oder Notwendigkeit?: - Die Rolle des Geschlechts in der Medizin. XX.

[9] Berg M, Appelman Y, Bekker MHJ, Toppen A (2015). Gender and Health. Knowledge Agenda.

[10] Ludwig S, Dettmer S, Peters H, Kaczmarczyk G (2016). Geschlechtsspezifische Medizin in der Lehre – noch in den Kinderschuhen. Dtsch Ärztebl.

[11] Burghaus D, Becker JC, Kappes K, Heue M, Kindler-Rohrborn A, Pfleiderer B (2016). Geschlechtsspezifisches Wissen und Gendersensibilitat in der medizinischen Lehre - eine Bestandsaufnahme. Gesundheitswesen.

[12] Berger M, Störmann S, Fischer MR (2007). Eine studentische Wiki-Bibliothek für unterrichtsbegleitende Materialien: Konzeption, Implementierung und Evaluation für das Medizinische Curriculum München (MeCuM). GMS Z Med Ausbild.

[13] Huwendiek S, Muntau AC, Maier EM, Tönshoff B, Sostmann K (2008). E-Learning in der medizinischen Ausbildung. Monatsschr Kinderheilkd.

[14] Augar N, Raitman R, Zhou W, Atkinson R, McBeath C, Jonas-Dwyer D, Phillips R (2004). Teaching and learning online with wikis. Beyond the comfort zone: Proceedings of the 21st ASCILITE Conference.

[15] Rost J, Carstensen CH, von Davier M (1999). Sind die Big Five Rasch-skalierbar?: 1 1 Diese Arbeit entstand im Rahmen des Forschungsprojektes "Mischverteilungsmodelle" am IPN - Institut für die Pädagogik der Naturwissenschaften - Kiel mit Unterstützung der Deutschen Forschungsgemeinschaft unter dem Titel RO 665-4. Diagnostica.

[16] Müller FH (2001). Studium und Interesse: Eine empirische Untersuchung bei Studierenden.

[17] Deci EL, Ryan RM (2000). The "What" and "Why" of Goal Pursuits: Human Needs and the Self-Determination of Behavior. Psychol Inquiry.

[18] Verdonk P, Mans LJL, Lagro-Janssen ALM (2005). Integrating gender into a basic medical curriculum. Med Educ.

[19] Ludwig S, Oertelt-Prigione S, Kurmeyer C, Gross M, Gruters-Kieslich A, Regitz-Zagrosek V, Peters H (2015). A Successful Strategy to Integrate Sex and Gender Medicine into a Newly Developed Medical Curriculum. J Womens health (Larchmt).

