# Supplementary material for: Development and initial Experience of an online Exchange Platform on Sex and Gender Aspects in Medicine: “GenderMed-Wiki”
Source: GMS J Med Educ. 2018 Aug 15;35(3):Doc32. doi: 10.3205/zma001178 (PMC6120162; doi:10.3205/zma001178)
Supplement: Minutes of the focus groups: four categories were identified which needed further optimization. Suggestions that were not implemented prior to the evaluation of the platform are marked in italic and bold. [file JME-35-32-s-001.pdf]

Aspects related to content

1. The layout of the pages should be altered:
   - An abstract and a general introductory easy to understand article (fact sheet) should be included as introduction to the topic for users. The hitherto differentiation between lay and expert article should not be continued.
2. Since technical terms have not been explained so far, it was suggested to add a glossary:
   - A glossary with technical terms will be added with an own menu item. Technical terms in articles and corresponding explanation in the glossary will be linked automatically to foster a better understanding of the articles.
   - “GenderMed-Wiki” will implement a glossary with technical terms. In the future, if an author drafts an article, already existing technical terms will be automatically linked with the glossary. In case technical terms are missing authors should send an e-mail to the editorial team to ask them to add the missing term.
3. It was suggested to add a knowledge quiz at the end of each article to assess one´s knowledge. Mediawiki has a template which can be used for quizzes.
4. It is of major importance to use sex and gender sensitive language (e.g. use of sex/gender sensitive medicine, but terms such as or sex/gender specific or sex/gender equitable medicine should be avoided)
5. Optimization of graphic elements in the article: e.g. coloring of subheading, outlines, box with categories, tables and frames around pictures. The logo of “GenderMed-Wiki” should be evaluated in the online survey and altered accordingly.
6. It was suggested to add/amend the following items in the outline of article in a standardized way:

- The item “outlook” will be changed into “open research questions”.
- A new item will be added: Integration into patient-centered care“. This should improve the clinical usability of the platform.

7. ***New articles as well as the best rated article of the month should be highlighted on the main page of “GenderMed-Wiki“.***
8. “GenderMed-Wiki“ is not a knowledge portal which explains symptoms of diseases in an easy way. This needs to be more clearly pointed out on the webpage.
9. ***The possible number of subjects (e.g. psychiatry) associated with an article should be increased from 3 to 5.***
10. There are not enough pictures and graphs in the articles. This makes it a hard reading. More graphs and pictures will be included in existing articles.
11. ***Sources should be better described (What type of source is this? e.g. meta-analysis, scientific report.), this will bring additional value to the source.***
12. Authors and members of the review-board much be informed that redundancies in the articles cannot be avoided, since each menu item should be understandable on its own. Not every user may be reading the complete article.

Technical requirements

1. Introductory and professional articles should be available as pdf for downloading
2. ***Implementation of a RSS-Web-Feed-function:***
   - ***It should be possible to subscribe for news alerts with the latest information on the platform by e-mail (e.g. a new article just went online) already at registration for “GenderMed-Wiki” by ticking the corresponding box.***
   - ***The menu item “Infothek” should inform users that it is possible to subscribe to an e-mail alert by an e-mail to the editor. To unsubscribe from the news-alert should be possible in an easy way as well.***
   - ***An e-mail alert about new content should be sent out every two weeks***
3. ***The editor should be informed automatically by e-mail of outdated articles which are older than 5 years to ensure up-to-date information and not to lose track.***
4. ***Articles and teaching materials should be accessible by a filter function either manually or by a drop-down list.***

Usability of the platform

1. ***It is planned to establish “GenderMed-Wiki“ as elective course at the medical faculty Muenster. Part of the academic assessment could be drafting an article for this platform. If this is proven to be suitable format, this could be offered to other medical faculties.***
2. ***A German-wide competition could be organized at the 1th anniversary of the platform. The article with the highest rating could be given an award***
3. ***General practitioners could be invited to contribute a case study. It is of importance to anonymize all data to protect the privacies of patients.***
4. ***In the long run an “exchange platform for cooperation” e.g. for students might be of interest.***
5. ***Articles can only keep up-to-date by increased participation of the “scientific community”. For example articles for “GenderMed-Wiki” could be part uses as an obligatory qualification paper when doing a doctoral thesis with a focus on sex/gender sensitive medicine.***

Legal challenges

1. ***In is of high importance to explain legal aspects in a transparent way. Users of the platform must understand how to use the contents of platform legally in a correct way.***
2. ***It should be possible that passive users can access the platform freely and do not need to register to read articles. Active users (authors, review- and editorial-board members) must register before being able to draft or amend/change articles, as well when drafting/changing and using teaching materials.***
3. The editorial-board serves as higher authority to overview of the legal aspects.
4. ***The name of authors of articles must not be given, it is voluntarily. Nevertheless it must be transparent if articles have been amended or altered and the date when this was done. Altering or amending articles by others is the main principle of a Wiki and must be accepted by authors. If an author wants to reduce the risk that an article published under his/her name is altered substantially by others, he/she needs to publish his/her article anonymously.***
5. It is of importance to review all articles after amendments or changes to ensure high scientific quality..
